# Genetic modifiers of hypertension in sGC-deficient mice

**DOI:** 10.1186/2050-6511-14-S1-O8

**Published:** 2013-08-29

**Authors:** Emmanuel Buys

**Affiliations:** 1 Anesthesia Center for Critical Care Research Department of Anesthesia, Critical Care, and Pain Medicine, Massachusetts General Hospital, Harvard Medical School, USA

## Background

Hypertension is an important modifiable risk factor for coronary heart disease, congestive heart failure, stroke, end-stage renal disease, and peripheral vascular disease. Many of the molecular mechanisms and genetic factors underlying the development of the most common forms of human hypertension remain to be defined. Nitric oxide (NO) and one of its primary targets, the cyclic guanosine monophosphate (cGMP) generating enzyme soluble guanylate cyclase (sGC), play an essential role in regulating blood pressure, in part by by modulating relaxation of vascular smooth muscle.

## Results

Male mice deficient in the α1 subunit of soluble guanylate cyclase (sGCα1-/- mice) are prone to hypertension in some, but not all, mouse strains, suggesting that additional genetic factors contribute to the onset of hypertension associated with sGCα1-deficiency. Using linkage analyses, we discovered quantitative trait loci (QTL) that were linked to mean arterial pressure (MAP) in sGCα1-/- mice. One locus is syntenic with previously identified blood pressure–related QTLs in the human and rat genome and contains the genes coding for renin. Hypertension in sGCα1-/- mice was associated with increased activity of the renin-angiotensin-aldosterone system (RAAS) and RAAS inhibition normalized MAP and improved endothelium-dependent vasorelaxation in sGCα1-/- mice.

## Conclusion

These data identify the RAAS as a blood pressure–modifying mechanism in a setting of impaired NO/cGMP signaling [1].
